# LIGHT aggravates sepsis‐associated acute kidney injury via TLR4‐MyD88‐NF‐κB pathway

**DOI:** 10.1111/jcmm.15815

**Published:** 2020-09-03

**Authors:** Yu Zhong, Shun Wu, Yan Yang, Gui‐qing Li, Li Meng, Quan‐you Zheng, You Li, Gui‐lian Xu, Ke‐qin Zhang, Kan‐fu Peng

**Affiliations:** ^1^ Department of Nephrology Southwest Hospital Army Medical University Chongqing China; ^2^ Department of Immunology Army Medical University Chongqing China; ^3^ Department of Urology 958 Hospital Southwest Hospital Army Medical University Chongqing China; ^4^ Department of intense care Daping Hospital Army Medical University Chongqing China; ^5^ Urinary Nephropathy Center The Second Affiliated Hospital of Chongqing Medical University Chongqing China

**Keywords:** acute kidney injury, LIGHT, NF‐κB, sepsis, TLR4

## Abstract

Sepsis‐associated acute kidney injury (SA‐AKI) is a common clinical critical care syndrome. It has received increasing attention due to its high morbidity and mortality; however, its pathophysiological mechanisms remain elusive. LIGHT, the 14th member of the tumour necrosis factor (TNF) superfamily and a bidirectional immunoregulatory molecule that regulates inflammation, plays a pivotal role in disease pathogenesis. In this study, mice with an intraperitoneal injection of LPS and HK‐2 cells challenged with LPS were employed as a model of SA‐AKI in vivo and in vitro, respectively. LIGHT deficiency notably attenuated kidney injury in pathological damage and renal function and markedly mitigated the inflammatory reaction by decreasing inflammatory mediator production and inflammatory cell infiltration in vivo. The TLR4‐Myd88‐NF‐κB signalling pathway in the kidney of LIGHT knockout mice was dramatically down‐regulated compared to the controls. Recombinant human LIGHT aggravated LPS‐treated HK‐2 cell injury by up‐regulating the expression of the TLR4‐Myd88‐NF‐κB signalling pathway and inflammation levels. TAK 242 (a selective TLR4 inhibitor) reduced this trend to some extent. In addition, blocking LIGHT with soluble receptor fusion proteins HVEM‐Fc or LTβR‐Fc in mice attenuated renal dysfunction and pathological damage in SA‐AKI. Our findings indicate that LIGHT aggravates inflammation and promotes kidney damage in LPS‐induced SA‐AKI via the TLR4‐Myd88‐NF‐κB signalling pathway, which provide potential strategies for the treatment of SA‐AKI.

**Clinical Perspectives**

- The role of LIGHT on SA‐AKI has never been reported.
- Our results indicate that LIGHT aggravates LPS‐induced SA‐AKI via up‐regulating the TLR4‐Myd88‐NF‐κB pathway expression.
- The results allow us to further understand the effects of LIGHT in SA‐AKI to develop novel therapies.

## 1 INTRODUCTION

Sepsis is a life‐threatening syndrome caused by a dysregulated host response to infection.^1^, ^2^ Sepsis most commonly results in multiorgan dysfunction, especially kidney dysfunction, namely sepsis‐associated acute kidney injury (SA‐AKI). It constitutes almost 50% of cases diagnosed with acute kidney injury in intensive care units (ICU).^3^, ^4^, ^5^, ^6^, ^7^ Furthermore, SA‐AKI increases the risk of chronic kidney disease and is a leading independent cause of high mortality.^2^ Unlike any other phenotype of acute kidney injury (AKI), SA‐AKI has a convoluted and exclusive pathophysiology, which is not fully understood. Recent evidence shows that inflammation, oxidative stress, disturbances in coagulation and the adaptive response of renal tubular epithelial cells to injury may contribute to the development of SA‐AKI.^1^, ^2^, ^8^, ^9^, ^10^, ^11^

LIGHT (Homologous to Lymphotoxins, exhibits inducible expression and competes with HSV Glycoprotein D for HVEM, a receptor expressed by T lymphocytes), the 14th member of the TNF superfamily, has been identified as a novel immunoregulatory molecule.^12^, ^13^ LIGHT signals by combining its two receptors, the Herpes Virus Entry Mediator (HVEM) and the lymphotoxin β receptor (LTβR), may play a bidirectional regulatory role in inflammatory disorders.^14^, ^15^, ^16^, ^17^ By interacting with HVEM, LIGHT signalling can produce a co‐stimulatory signal for the activation and proliferation of T cells and cause the production of cytokines and inflammatory factors to promote proinflammatory responses.^13^, ^18^ Meanwhile, the LIGHT‐LTβR pathway may also increase the release of chemokines and adhesion molecules to induce immune cell recruitment to accelerate immunoreaction.^19^

A range of studies have demonstrated that the LIGHT pathway plays an important role in the pathophysiology of several inflammatory diseases, including rheumatoid arthritis, IgA nephropathy and inflammatory bowel disease.^14^, ^15^, ^16^, ^17^ However, the effect of LIGHT on acute kidney injury has not yet been reported. Our study demonstrated that LIGHT deficiency significantly attenuated SA‐AKI via the TLR4‐MyD88‐NF‐κB pathway, suggesting that LIGHT may act as an innovative intervention target in the pathogenesis of SA‐AKI.

## 2 MATERIALS AND METHODS

### 2.1 Mice and SA‐AKI model

All animal experiments were performed with the approval of the Laboratory Animal Welfare and Ethics Committee of the Army Medical University. LIGHT knockout (LIGHT KO) mice (C57BL/6J background) were donated by Professor Klaus Pfeffer (University of Düsseldorf, Dusseldorf, Germany). Wild‐type (WT) mice (C57BL/6J background, SCXK019‐0010) were obtained from SPF Biotechnology Co., Ltd. (Beijing, China). Male mice (weight 22‐25 g, 6‐8 weeks old) were used in all experiments. The mice were fed in an animal care facility at a stationary temperature of 23 ± 2°C with free access to food and water. Mice were intraperitoneally injected LPS (*Escherichia coli* 055:B5; Sigma‐Aldrich, St. Louis, USA) to establish a model of SA‐AKI in vivo, as previously described.^20^ Briefly, mice were administered a single intraperitoneal injection of LPS (20 mg/kg, LPS diluted in 0.9% normal saline, n = 6). The control mice were injected with saline solution (n = 6). After 24 hours from the injection of LPS or 0.9%, the mice were killed and blood samples and kidney tissues were collected. The kidneys were snap‐frozen in liquid nitrogen and stored at ‒80°C until total RNA or protein extraction. The kidneys were immediately embedded in 4% paraformaldehyde for haematoxylin and eosin (H&E) staining, immunohistochemistry (IHC) and immunofluorescence (IF). In addition, the dosage of the intraperitoneal injection was adjusted to 40 mg/kg for the survival study.^20^ Briefly, the WT and LIGHT KO mice were we divided into four groups after LPS or 0.9% saline injection: WT + LPS group (n = 13), LIGHT KO + LPS group (n = 13), WT + Saline group (n = 11), and LIGHT KO + Saline group (n = 11). Mouse survival was monitored every 6 hours for a total of 4 days (96 hours).

### 2.2 Cell culture

The human kidney tubular epithelial cell line (HK‐2 cell) was donated by Professor Yani He, who bought it from the American Type Culture Collection (Manassas, VA, USA). The cells were cultured in DMEM/F12 supplemented with 10% foetal bovine serum (FBS) (Gibco, New Zealand) and antibiotics (100 IU/mL penicillin and 100 mg/mL streptomycin) in a humidified atmosphere of 5% CO_2_ and 95% O_2_ at 37°C.

### 2.3 Cell viability assay and treatment

The HK‐2 cells were plated in 96‐well plates at a density of 5 × 10^3^ cells/mL per well for 24 hours. The cells were challenged with various concentrations of LPS (1, 5, 10, 25, 50 and 100 µg/mL) for 24 hours determine the optimal concentration of LPS for inducing cell injury. Similarly, HK‐2 cells were treated with recombinant human LIGHT (rhLIGHT) (0, 0.1, 0.25, 0.5, 1 and 5 µg/mL) with or without LPS for an additional 24 hours (data not shown) to determine the optimal concentration for aggravating or diminishing cell injury. Cell viability was determined using a Cell Counting Kit‐8 (CCK‐8) assay kit (Bioss, Beijing, China). The absorbance of the different groups was assayed at 450 nm using a spectrophotometer (Bio‐Rad, USA). Then, the HK‐2 cells were incubated with serum‐free DMEM/F12 for 12 hours and divided into four groups before culturing for another 24 hours as follows: (a) vehicle group, (b) LPS group (50 µg/mL), (c) LPS (50 µg/mL) + rhLIGHT (5 µg/mL), (d) LPS (50 µg/mL) + rhLIGHT (5 µg/mL) + TAK242 (0.5 µmol/L). TAK242 (MedChemExpress, Monmouth Junction, NJ, USA), a TLR4 inhibitor, was tested for blocking the effect of LIGHT on SA‐AKI in vitro. After 2 hours of pretreatment with TAK242, the HK‐2 cells were challenged with LPS and rhLIGHT simultaneously. The vehicle groups were challenged with an equal volume of vehicle.

### 2.4 Cell apoptosis assay

After treatment, the HK‐2 cells were harvested. Apoptosis was assessed by flow cytometry following the manufacturer's instructions (Cwbiotech, China).

### 2.5 Measurement of Scr and BUN levels in plasma

Blood samples were obtained from the periorbital sinus under anaesthesia. All mice were killed by cervical dislocation at 24 hours after LPS or 0.9% saline injection. The levels of serum creatinine (SCr) and blood urea nitrogen (BUN) were determined using a biochemical autoanalyser (Olympus AU5400; Olympus, Japan) to evaluate renal function, following the manufacturer's instructions.

### 2.6 ELISA

The concentrations of TNF‐α, IL‐6 and IL‐1β in serum were determined using ELISA kits (USCN Life Science Inc, China) following the manufacturer's instructions. The optical density (OD) was determined at 450 nm using a spectrophotometer (Bio‐Rad, USA). The expression levels of TNF‐α, IL‐6 and IL‐1β were calculated from a standard curve.

### 2.7 Histological evaluation

After fixation in 4% paraformaldehyde overnight, the tissue from the right kidney was embedded in paraffin, sectioned and stained with H&E for observation under a light microscope (Olympus BX63; Olympus, Japan). Histological evaluation was performed by two experienced doctors in a double‐blind manner. The 0‐4 semi‐quantitative scales were employed in the assessment, as previously described.^21^, ^22^ Ten random fields (400×) of cortical tissues in every mouse were counted and the percentage of injured area was determined. Tissue damage was scored according to the percentage of damaged tubules: 0, no damage; 1, <25%; 2, 25‐50%; 3, 51‐75%; 4, >75%.^21^, ^22^

### 2.8 MPO assay

Renal tissue samples were mixed with buffer solution (1:19) to prepare 5% homogenate using a tissue homogenizer (Jingxin, Shanghai, China). To evaluate the accumulation of neutrophils, the MPO concentration was determined using commercial reagent kits (Jiancheng, Nanjing, China) following the manufacturer's instructions.

### 2.9 RNA extraction and quantitative real‐time PCR

Total RNA from kidney tissues and HK‐2 cells was extracted using TRIzol reagent (TaKaRa Bio, Japan) according to the manufacturer's protocols. A NanoDrop spectrophotometer (ND‐100; Thermo Scientific, USA) was used to assay the RNA ºconcentration. After reverse transcription, the target gene expression was determined by quantitative real‐time PCR using SYBR Premix Ex Taq II kits (TaKaRa Bio, Japan) following the manufacturer's instructions in a PCR system (Mx3000; Stratagene, USA). As shown in Table 1, the primer sequences were synthesized by Invitrogen Co., Ltd. (Shanghai, China). Target gene expression was standardized with GAPDH and calculated using the 2^−∆∆CT^ method.

**Table 1:** Sequences of the primers for real‐time PCR

Gene	Sequence(5′‐>3′)
F‐GAPDH(mouse)	AGGTCGGTGTGAACGGATTTG
R‐GAPDH(mouse)	TGTAGACCATGTAGTTGAGGTCA
F‐KIM‐1(mouse)	ACATATCGTGGAATCACAACGAC
R‐KIM‐1(mouse)	ACAAGCAGAAGATGGGCATTG
F‐NGAL(mouse)	TGGCCCTGAGTGTCATGTG
R‐NGAL(mouse)	CTCTTGTAGCTCATAGATGGTGC
F‐TNF‐α(mouse)	CCTTATCTACTCCCAGGTTCTC
R‐TNF‐α(mouse)	GAGGCTGACTTTCTCCTGGTATG
F‐IL‐6(mouse)	GCCCTTCAGGAACAGCTATGA
R‐IL‐6(mouse)	TGTCAACAACATCAGTCCCAAGA
F‐MCP‐1(mouse)	TTAAAAACCTGGATCGGAACCAA
R‐MCP‐1(mouse)	TTAAAAACCTGGATCGGAACCAA
F‐ICAM‐1(mouse)	GTGATGCTCAGGTATCCATCCA
R‐ICAM‐1(mouse)	CACAGTTCTCAAAGCACAGCG
F‐GAPDH(human)	GGCTGTTGTCATACTTCTCATGG
R‐GAPDH(human)	GGAGCGAGATCCCTCCAAAAT
F‐IL‐6(human)	ACTCACCTCTTCAGAACGAATTG
R‐IL‐6(human)	CCATCTTTGGAAGGTTCAGGTTG
F‐IL‐1β(human)	GTCGGAGATTCGTAGCTGGA
R‐IL‐1β(human)	ATGATGGCTTATTACAGTGGCAA
F‐TNF‐α(human)	CCTCTCTCTAATCAGCCCTCTG
R‐TNF‐α(human)	GAGGACCTGGGAGTAGATGAG
F‐LIGHT(mouse)	TGGCTCCTGTAAGATGTGCTG
R‐LIGHT(mouse)	GTTTCTCCTGAGACTGCATCAA
F‐LTBR(mouse)	TGCATACCGCAAAGACAAACTC
R‐LTBR(mouse)	TGGTGCCCCCTTATCGCATA
F‐HVEM(mouse)	ACTCGTCTCCCACAAGGAACT
R‐HVEM(mouse)	CAGGCCCCTACAGACAACAC

### 2.10 Western blot analysis

The total proteins of the kidney tissues and HK‐2 cells were lysed and extracted using T‐PER^™^ Tissue Protein Extraction Reagent (Thermo Fisher, USA) following the manufacturer's instructions. The protein concentration was assessed using the Enhanced BCA Protein Assay Kit (Beyotime Biotechnology, China). 8%, 10% and 12% SDS‐PAGE gels were prepared to separate proteins according to the molecular weight of the target proteins. The proteins were transferred onto a PVDF membrane (Millipore, USA) after electrophoresis. The membranes were incubated with 5% of bovine serum albumin at room temperature for 1 hour to block non‐specific binding. Different concentrations of primary antibodies against TLR4 (1:1000; Abcam), MyD88 (1:1000; CST), NF‐κB P65 (1:1000; CST), p‐NF‐κB P65 (1:1000; CST), LIGHT (1:1000; Abcam), HVEM (1:500; Santa Cruz), LTβR (1:1000; Abcam), tubulin (1:2000; Beyotime) and GAPDH (1:2000; Bioworld) were prepared and incubated at 4°C overnight. Then, the secondary antibody (1:2000; CST) was prepared to incubate the membranes at room temperature for 1 hour according to the species of the primary antibody. Finally, an enhanced chemiluminescence reagent (ECL) (Millipore, USA) was used to visualize the immunoreactive bands in a gel imaging and analysis system (Vilber Fusion Solo, France).

### 2.11 Immunohistochemistry

After fixation in 4% paraformaldehyde overnight, the kidney tissue was embedded in paraffin, sectioned and incubated in primary antibodies against LIGHT (1:200; Abcam), HVEM (1:200; Santa Cruz), LTβR (1:200; Abcam) and TLR4 (1:200; Abcam) overnight at 4°C. Then, the stained tissues were incubated with secondary peroxidase‐conjugated antibodies (1:2000; Wanleibio) at room temperature for 1 hour according to the species of the primary antibodies. Lastly, the kidney sections were observed under a light microscope (Olympus BX63; Olympus, Japan) after DAB staining.

### 2.12 Immunofluorescence

For immunofluorescence staining, formalin‐fixed tissue slices were incubated with the following primary antibodies: Ly6G (1:200; Abcam), F4/80 (1:200; Abcam), LIGHT (1:200; Bioss), LTβR (1:200; Bioss), HVEM (1:200; Santa Cruz), plus CK‐18 (1:200; Bioss) overnight at 4°C, followed by secondary peroxidase‐conjugated antibodies under dark conditions for 1 hour. To determine the cellular localization of LIGHT and its receptors, the co‐localized expression of LIGHT‐LTβR/HVEM and CK‐18 was assessed by confocal laser scanning microscopy (TCS SP5; Leica, Germany).

According to the group allocation, the HK‐2 cells were treated as described above. The cells were then stained with antibodies against LIGHT (1:200; Bioss), HVEM (1:100; Santa Cruz), LTβR (1:200; Abcam) and p‐NF‐κB‐P65 (1:100) overnight. The cells were incubated with a FITC‐labelled secondary antibody (200:1; BioLegend) and Hoechst33258 (300:1; Sigma) for visualization, according to the manufacturer's instructions. After immunostaining, the immunostained images were captured using a fluorescent microscope (BX63; Olympus, Japan) at 200 × magnification. To observe the p‐NF‐κB‐P65 nuclear translocation, immunofluorescence detection of p‐NF‐κB‐P65 was performed using a laser scanning confocal microscope (TCS SP5; Leica, Germany).

### 2.13 In vivo LIGHT blocking experiments

WT mice were randomly divided into four groups (n = 6): (a) control group, (b) LPS + Ig‐Fc group, (c) LPS + HVEM‐Fc group and (d) LPS + LTβR‐Fc group. Mice were pretreated by injecting intraperitoneally with LTβR‐Fc (100 µg) (the murine extramembrane LTβR fused with human IgG Fc fragment, Zoonbio Biotechnology Co.) or HVEM‐Fc (100 µg) (the murine extramembrane HVEM fused with human IgG Fc fragment, Sino Biological Inc) for 24 and 2 hours of LPS administration depending on the group. Renal function and pathological damage were assessed and compared as previously described.

### 2.14 Statistical analysis

All data are presented as the mean ± standard error of the mean (SEM) from at least three independent experiments. GraphPad Prism 6.0 (La Jolla, California, USA) was used for statistical analysis. Student's *t* test was used to compare two groups, whereas the intergroup differences were analysed using one‐way ANOVA with Dunnett's multiple comparisons tests. A *P*‐value of 0.05 was considered statistically significant.

## 3 RESULTS

### 3.1 Sepsis increased LIGHT expression in LPS‐induced SA‐AKI

**Figure 1:**
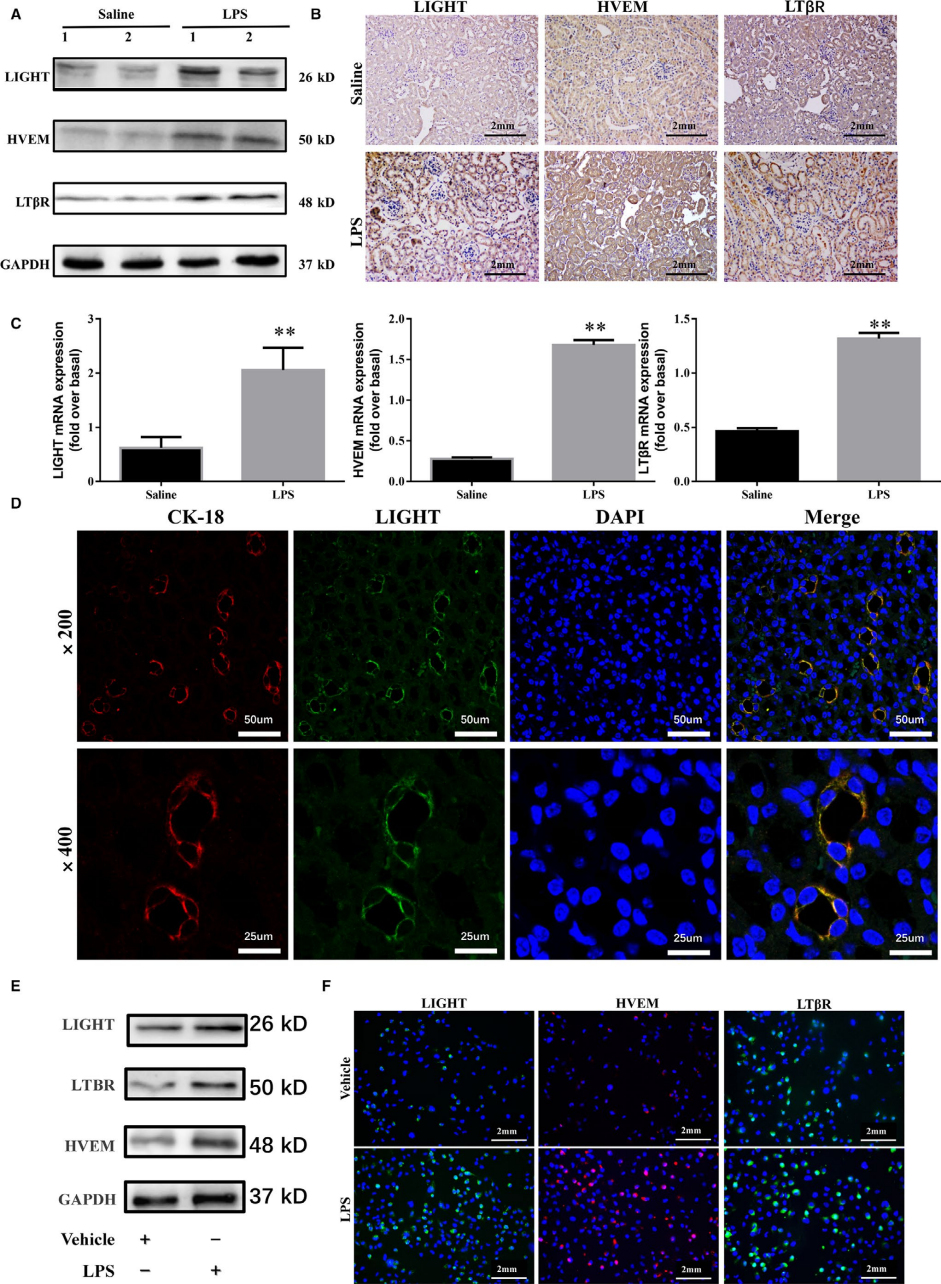
Sepsis increased LIGHT expression in LPS‐induced SA‐AKI. A, Western blot for the expression of LIGHT, HVEM and LTβR in kidney tissues from WT mice at 24 h after LPS (20 mg/kg) or saline injection. Western blots are of three independent experiments. B, Representative immunohistochemistry images of LIGHT, HVEM and LTβR of kidney tissues from WT mice at 24 h after LPS (20 mg/kg) or saline injection. C, LIGHT, HVEM and LTβR mRNA levels were determined by quantitative RT‐PCR in the kidney of WT or LIGHT KO mice at 24 h after LPS or saline injection ± SEM, *P < 0.05. **P < 0.01, (n = 6). D, Immunofluorescence co‐localization of LIGHT and CK‐18. E, Western blot for the expression of LT, HVEM and LTβR in HK‐2 cells treated with or without LPS (50 μg/mL) for 24 h. F, Representative immunofluorescence images of LIGHT, HVEM and LTβR in HK‐2 cells treated with or without LPS (50 μg/mL) for 24 h (n = 6 for saline; n = 6 for LPS administration)

A wealth of research has demonstrated that proximal tubular epithelial cells serve as a primary target of sepsis.^20^, ^23^ To determine the role of LIGHT in SA‐AKI, we first examined the expression of LIGHT and its membrane‐bound receptors HVEM and LTβR on tubular epithelial cells in an in vivo and in vitro model of SA‐AKI, respectively. LIGHT and its receptor are constitutively expressed in renal tubular epithelial cells.^24^ In LPS‐injected mice, we observed a remarkable increase in the protein and mRNA levels of LIGHT, HVEM and LTβR in the kidney tissues compared with the saline‐injected control mice (Figure 1A‐C). The immunofluorescence results further demonstrated that LIGHT‐HVEM/LTβR expression was co‐localized with the expression of CK‐18, a marker of tubular epithelial cells (Figure 1D, Supplementary Figure S1A,B). Consistent with the in vivo findings, LPS challenge also induced a marked increase in LIGHT, HVEM and LTβR in HK‐2 cells in vitro (Figure 1E,F). These results demonstrate that LIGHT is closely related to LPS‐induced SA‐AKI, which forms the basis for our further study.

### 3.2 LIGHT deficiency prolonged the survival of SA‐AKI mice

**Figure 2:**
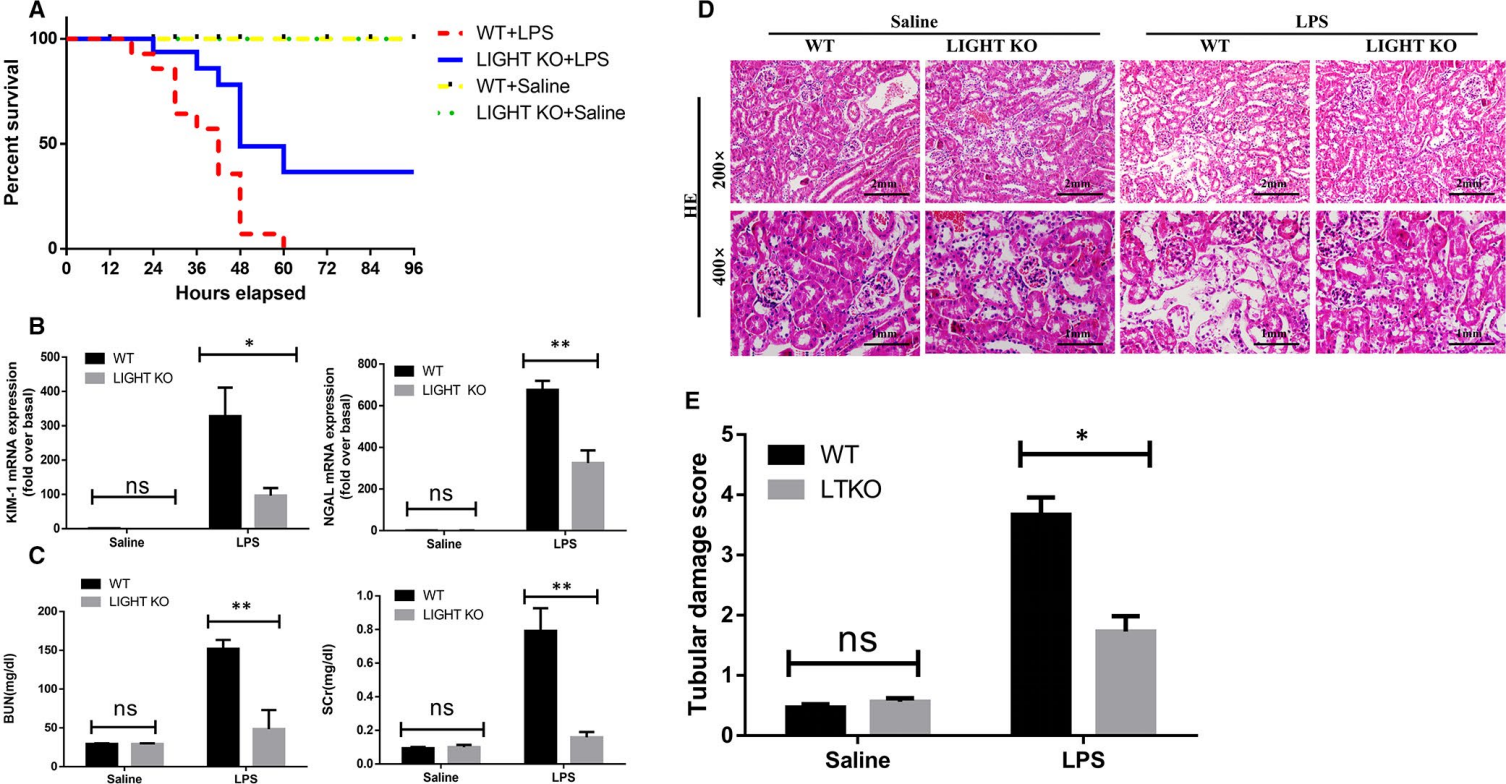
LIGHT deficiency protected against kidney tubular injury in SA‐AKI. A, Survival rate of WT and LIGHT KO mice after LPS (40 mg/kg, n = 13) or saline (n = 11) injection during the observation period. B, Renal injury markers KIM‐1mRNA and NGAL mRNA levels were determined by quantitative RT‐PCR in the kidney of WT or LIGHT KO mice at 24 h after LPS or saline injection ± SEM, *P < 0.05. **P < 0.01, (n = 6). C, Renal function measured by blood urea nitrogen and serum creatinine levels of WT and LIGHT KO mice at 24 h after LPS (20 mg/kg) or saline injection ± SEM, *P < 0.05 (n = 6). D, Representative images of H&E staining of tubular epithelial cells of kidney sections from WT or LIGHT KO mice at 24 h after LPS or saline administration. E, Semi‐quantitative analysis of tubular injury (loss of brush border, vacuolization and degeneration of renal tubular epithelial cells, tubular atrophy or dilatation, cast formation, cell lysis and inflammatory cell infiltration.) scored as: 0, no damage; 1, <25%; 2, 25‐50%; 3, 51‐75%; 4, >75% of affected area from ten random fields. The results are shown as the mean ± SEM. *P < 0.05. **P < 0.01 (n = 6)

To further elucidate the role of LIGHT in SA‐AKI, LIGHT KO mice and WT mice were injected with LPS (40 mg/kg, i.p.) or saline, and the survival rate was monitored every 6 hours for a total of 96 hours. As shown in Figure 2A, at the end of the experiment, all mice in the WT group died (n = 13) and 9 mice in the LIGHT KO group died (n = 13), whereas none of the mice in the saline treatment group died (n = 11). These findings suggest that LIGHT deficiency may play a protective role in a LPS‐induced sepsis model.

### 3.3 LIGHT deficiency protected against kidney injury in SA‐AKI mice in vivo

To further explore the protective effect of LIGHT on SA‐AKI, we investigated the degree of renal function and pathological injury. LPS caused a significant increase in the serum BUN and SCr levels in the model groups relative to the control groups. LIGHT KO mice showed ameliorated renal function, indicated by decreases in the expression levels of kidney injury molecule‐1 (KIM‐1), neutrophil gelatinase‐associated lipocalin (NGAL) (Figure 2B), BUN and SCr (Figure 2C). LPS resulted in some histopathological changes, including partial renal tubular epithelial vacuole degeneration or oedema, cell or protein casts, dilation of renal tubules, a narrowed lumen and interstitial telangiectasia. A 0 to 4 point scoring system was used to evaluate tissue injury.^21^, ^22^ Consistent with renal function, LIGHT KO mice exhibited milder renal pathological damage relative to WT mice after LPS injection (Figure 2D,E). These results demonstrate that LIGHT deficiency provides protection against kidney damage in SA‐AKI.

### 3.4 LIGHT deficiency decreased inflammatory mediator production and inflammatory cell infiltration in SA‐AKI in vivo

**Figure 3:**
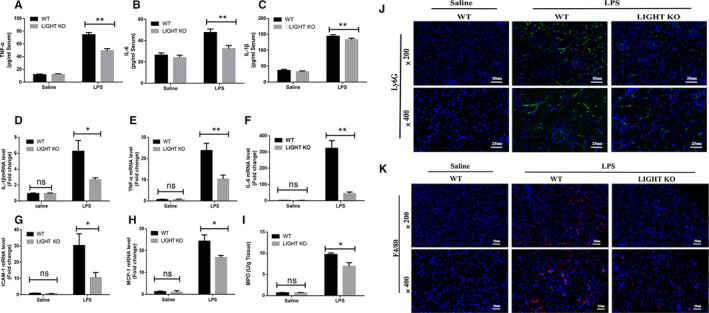
LIGHT deficiency alleviated inflammation in SA‐AKI in vivo. A‐C, The levels of TNF‐α, IL‐6 and IL‐1β in serum were determined by ELISA. D–H, The mRNA of IL‐1β (A), IL‐6 (B), TNF‐α (C), ICAM‐1 (D) and MCP‐1(E) in WT and LIGHT KO mice kidney tissues at 24 h after LPS (20 mg/kg) or saline injection was determined by quantitative RT‐PCR ± SEM, *P < 0.05. **P < 0.01 (n = 6). I, MPO activity in kidney tissues. J–K, Representative immunofluorescence images showing neutrophil (Ly6G) and monocyte‐macrophage (F4/80) infiltration in kidney tissue staining in WT and LIGHT KO mice kidney tissues at 24 h after LPS (20 mg/kg) or saline injection

A wealth of research has corroborated that inflammatory cell infiltration and proinflammatory cytokines, including TNF‐α, IL‐6 and IL‐1β, play a vital role in the pathological process of sepsis.^25^ Here, ELISA revealed that LIGHT deficiency down‐regulated LPS‐induced TNF‐α, IL‐6 and IL‐1β production in serum compared to WT mice (Figure 3A‐C). Similarly, LIGHT deficiency markedly inhibited LPS‐induced up‐regulation of TNF‐α, IL‐6, IL‐1β, ICAM‐1 and MCP‐1, according to quantitative RT‐PCR (Figure 3D‐H). As an indicator of neutrophilic infiltration in sepsis, the MPO activity in LIGHT KO mice was also remarkably attenuated compared to that in WT mice (Figure 3I). Consistent with the inflammatory cytokines, inflammatory cell infiltration showed a similar trend in LIGHT KO mice. Under the influence of ICAM‐1 and MCP‐1 production, polymorphonuclear neutrophils (PMN) and other phagocytic cells, such as monocyte macrophages, were down‐regulated in LIGHT KO mice compared to WT mice after LPS challenge (Figure 3J,K).

### 3.5 Exogenous LIGHT protein promoted cell injury in LPS‐treated HK‐2 cells

**Figure 4:**
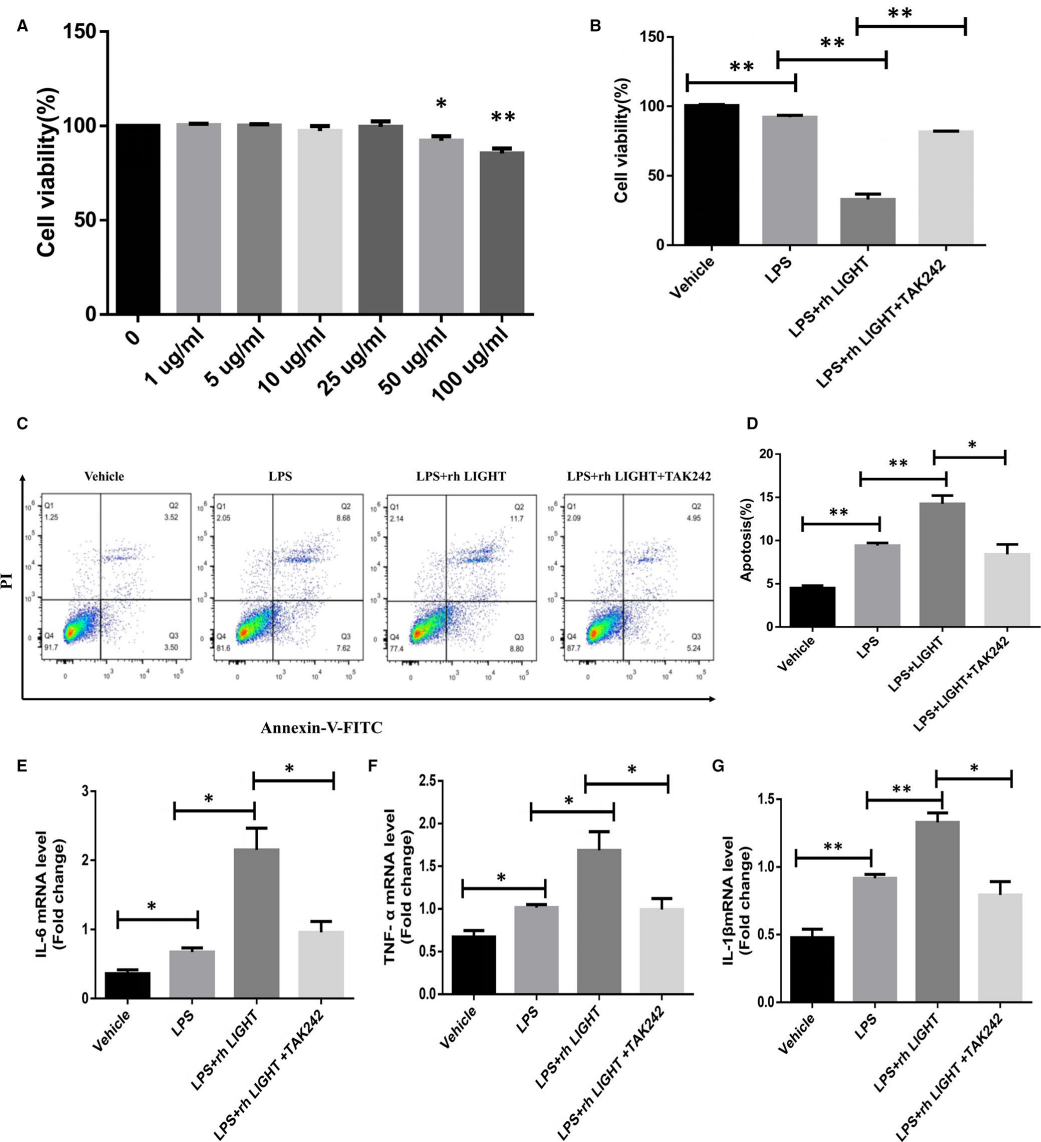
Exogenous LIGHT protein promotes cell injury in LPS‐treated HK‐2 cells. A, HK‐2 cells were treated in the absence or presence of various concentrations of LPS for 24 h. The resulting cell viability was determined by CCK‐8 assay. B, HK‐2 cells were pretreated with TLR4 inhibitor (TAK242) (0.5 μmol/L) and then incubated in the absence or presence of LPS (50 μg/mL) and rhLIGHT (5 μg/mL). CCK‐8 assay was performed at 24 h. C‐D, Analysis of apoptosis and apoptosis rate in HK‐2 cells by flow cytometry. E–G, Expression of TNF‐α, IL‐1β and IL‐6 was determined by quantitative RT‐PCR in HK‐2 cells. HK‐2 cells were pretreated with TLR4 inhibitor (TAK242) (0.5 μmol/L) and then incubated in the absence or presence of LPS (50 μg/mL) and rhLIGHT (5 μg/mL). The results are shown as the mean ± SEM. *P < 0.05. **P < 0.01

To further determine the effect of LIGHT on proximal tubular epithelial cells, recombinant human LIGHT protein (rhLIGHT) was used in LPS‐treated HK‐2 cells. After LPS challenge for 24 hours, the cell viability was found to decrease in a dose‐dependent manner in HK‐2 cells (Figure 4A). Treatment of the cells with LPS at 50‐100 µg/mL resulted in a decreased cell viability at 24 hours, whereas lower concentrations of LPS did not affect cell viability. As a result, 50 µg/mL was chosen as the minimal cytotoxic concentration of LPS. The optimal concentration of rhLIGHT was 5 µg/mL, according to a previous study^26^ and CCK‐8 assay (data not shown). The viability of HK‐2 cells in the LPS + rhLIGHT group decreased from 92.1% to 32.9% at 24 hours compared to the LPS group (Figure 4B). However, after TAK242 pretreatment, the cell viability of the LPS + rhLIGHT + TAK242 group recovered to 81.5% (Figure 4B). Flow cytometry showed that rhLIGHT aggravated the LPS‐induced apoptosis of HK‐2 cells, whereas TAK242 (a selective TLR4 inhibitor) largely reversed these effects (Figure 4C,D). In addition, we assessed the expression of inflammatory cytokines in HK‐2 cells after the treatment. Consistent with cell injury, the mRNA expression of IL‐6, IL‐1β and TNF‐α was markedly increased after LPS treatment, according to quantitative RT‐PCR (Figure 4E‐G). In addition, rhLIGHT and LPS significantly up‐regulated the expression of inflammatory cytokines. However, TAK242 inhibited the up‐regulation and decreased the expression of inflammatory mediators (Figure 4E‐G). These results indicate that LIGHT may promote cell injury via TLR4 signalling.

### 3.6 LIGHT deficiency down‐regulated TLR4‐MyD88‐NF‐κB signalling in SA‐AKI

**Figure 5:**
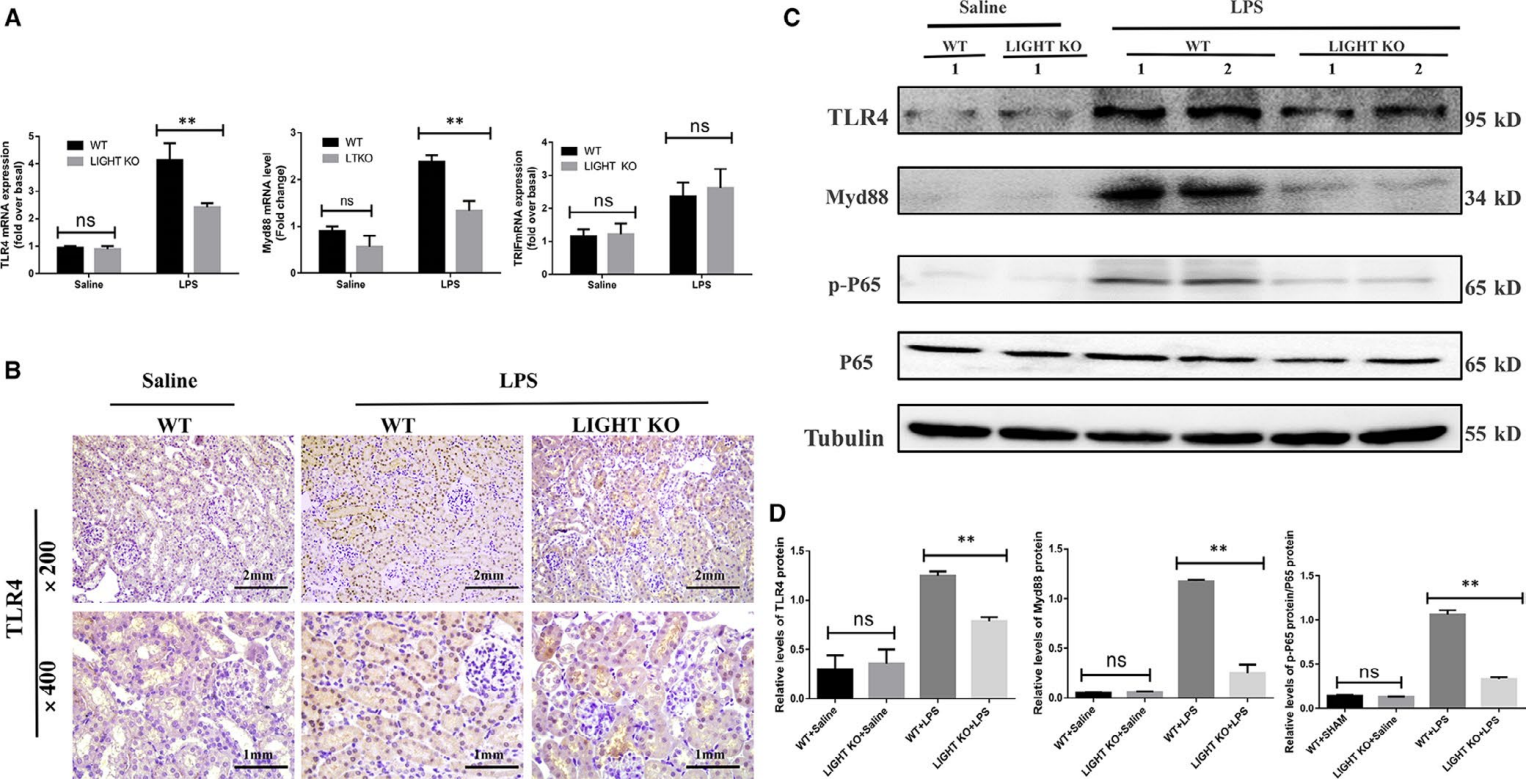
LIGHT deficiency inhibited TLR4‐myd88‐NF‐κB expression in SA‐AKI in vivo. A, The mRNA levels of TLR4, myd88 and TRIF in kidney tissues from WT or LIGHT KO mice at 24 h after LPS or 0.9% saline injection determined using quantitative RT‐PCR. B, Representative immunohistochemical images of TLR4 in kidney tissues from WT mice at 24 h after LPS (20 mg/kg) or saline injection. C–D, TLR4, myd88, NF‐κB and p‐NF‐κB expression in kidney tissues from WT or LIGHT KO mice at 24 h after LPS or saline injection detected by Western blotting. The results are shown as the mean ± SEM. *P < 0.05. **P < 0.01 (n = 6)

A myriad of studies has shown that the TLR4‐MyD88‐NF‐κB pathway is closely involved in the production of most inflammatory mediators induced by LPS, which can lead to the development of septic shock, multiple organ dysfunction and death.^11^, ^27^, ^28^ Therefore, we assessed the influence of LIGHT on the TLR4‐MyD88‐NF‐κB pathway using quantitative RT‐PCR. LIGHT deficiency was found to inhibit the LPS‐induced up‐regulation of TLR4 mRNA at the transcriptional level compared with WT mice at 24 hours after LPS injection (Figure 5A). Similarly, immunohistochemical results showed that LIGHT deficiency suppressed the LPS‐induced up‐regulation of TLR4 protein (Figure 5B). After the activation of TLRs, signals can be transmitted by two different signalling pathways: the MyD88‐dependent pathway and the TRIF‐dependent pathway.^29^ We also assessed the expression levels of TRIF mRNA, another downstream signal of TLR4, using quantitative RT‐PCR. However, no significant differences were observed in the TRIF mRNA levels between the LIGHT KO mice and WT mice in the model groups (Figure 5A). Intriguingly, MyD88 mRNA significantly decreased in the LIGHT KO mice compared to WT mice after LPS administration (Figure 5A), indicating that LIGHT may up‐regulate Myd88 as the main downstream pathway of TLR4 in the SA‐AKI model. Then, we determined whether TLR4 can activate the downstream signals of NF‐κB P65 through the MyD88‐dependent pathway. Western blotting showed that LIGHT deficiency suppressed the activation of TLR4‐MyD88‐NFκB protein in SA‐AKI in vivo compared to the WT mice (Figure 5C,D). Therefore, these results demonstrate that LIGHT deficiency significantly down‐regulates the expression of p‐NF

‐κB in the model groups.

**Figure 6:**
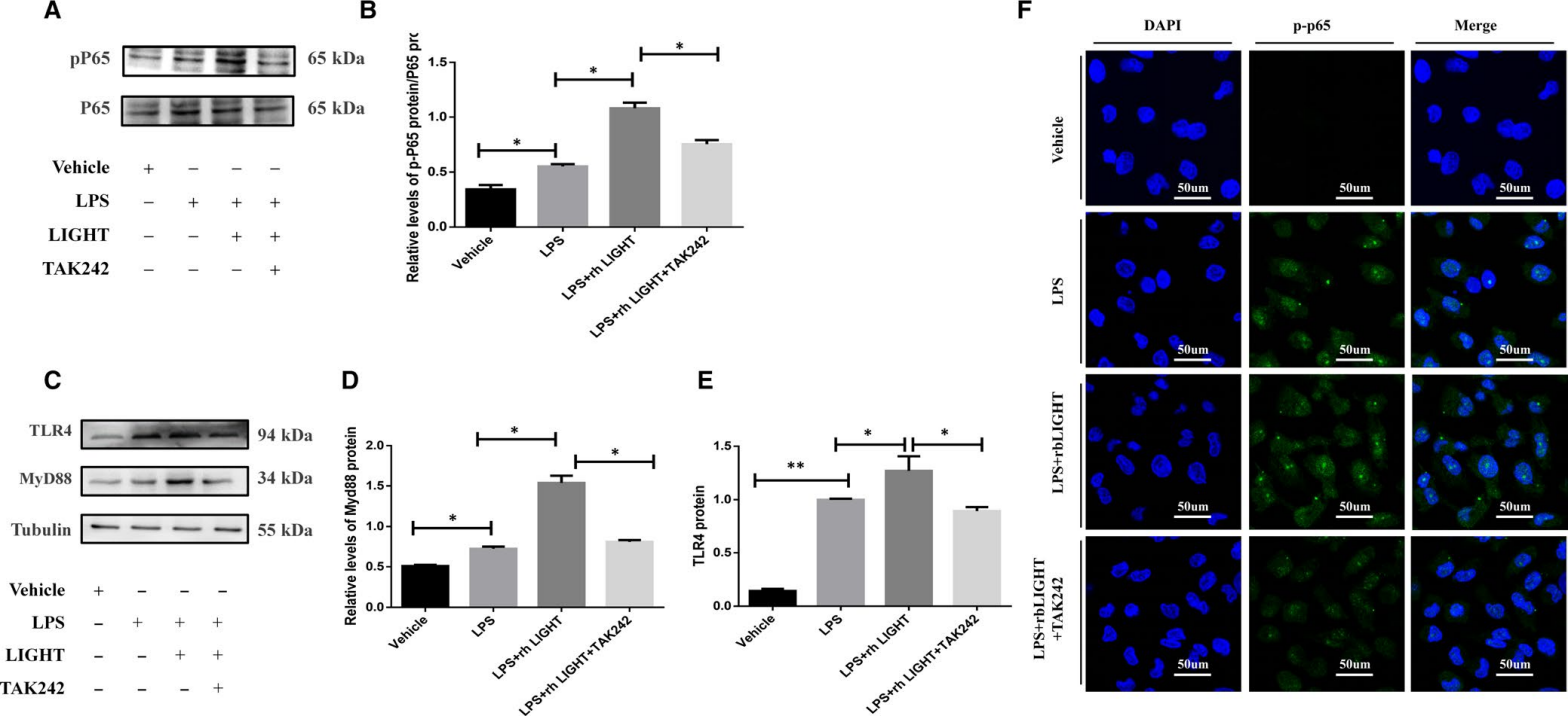
Exogenous LIGHT exacerbated HK‐2 cells via TLR4‐Myd88‐NF‐κB signalling. A‐E, TLR4, Myd88 and p‐NF‐κB expression in LPS‐treated HK‐2 cells with or without rhLIGHT detected by Western blotting. F, Effects of rhLIGHT on the nuclear translocation of NF‐κB in LPS treated with HK‐2 cells with or without rhLIGHT and TAK242 using immunofluorescence staining (magnification, 630×)

In accordance with the in vivo results, rhLIGHT directly promoted the expression of NF‐κB in LPS‐treated HK‐2 cells, whereas TAK242 pretreatment inhibited the increased expression of p‐NF‐κB/P65 (Figure 6A‐E). In addition, immunofluorescence staining analysis revealed that rhLIGHT facilitated NF‐κB/P65 translocation into the nucleus in HK‐2 cells, a role that was suppressed by TAK242 in vitro (Figure 6F). These results suggest that LIGHT deficiency mitigates LPS‐induced inflammatory signals via the TLR4‐MyD88‐NF‐κB pathway.

### 3.7 LIGHT blocking relieved SA‐AKI in vivo

**Figure 7:**
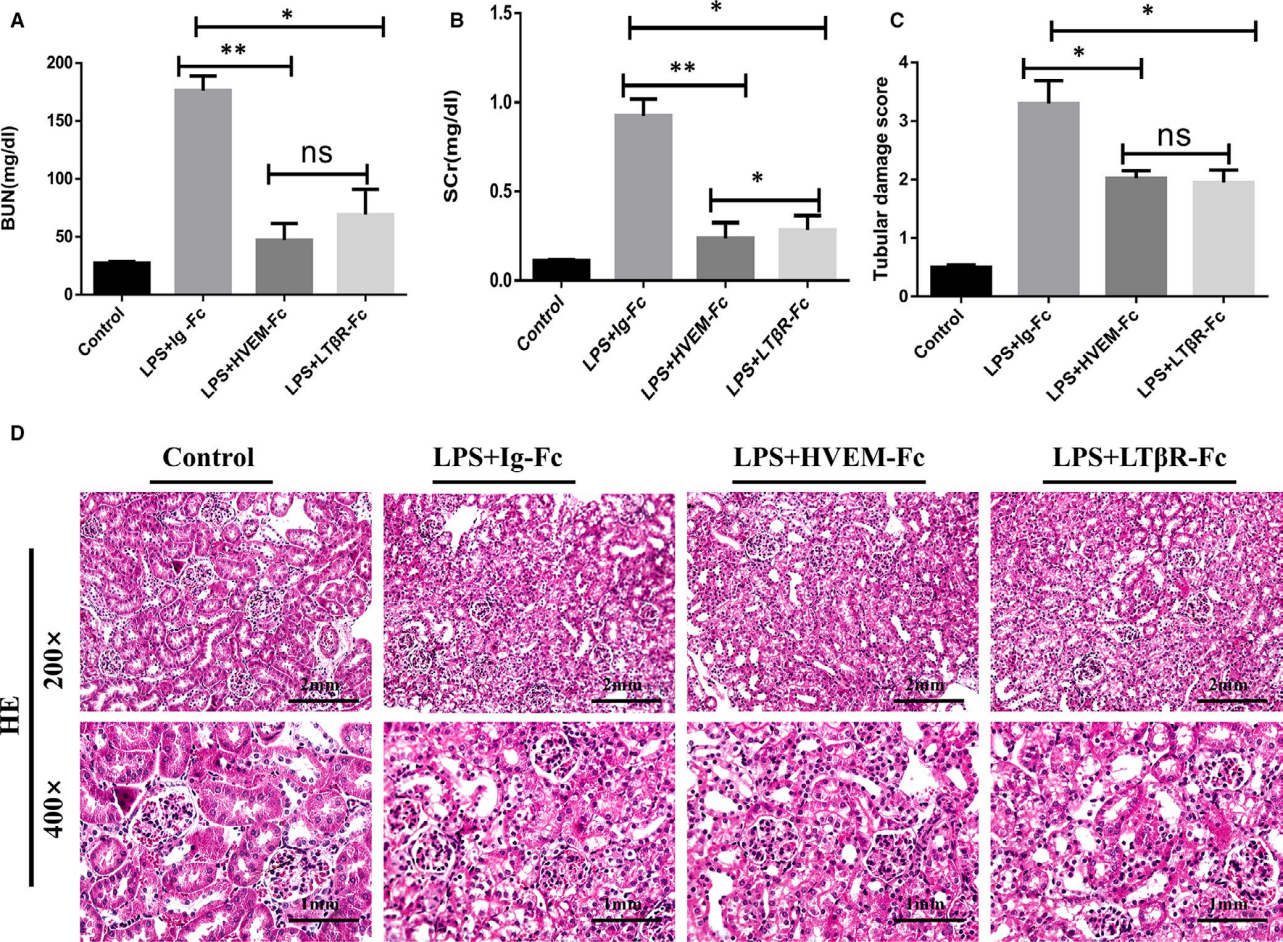
LIGHT blocking relieved SA‐AKI in vivo. A‐B, Renal function measured by blood urea nitrogen and serum creatinine levels of WT mice at 24 h after LPS (20 mg/kg) and HVEM‐Fc or LTβR‐Fc injection ± SEM, *P < 0.05 (n = 6). C, Semi‐quantitative analysis of tubular injury. D, Representative images of H&E staining of tubular epithelial cells of kidney sections from WT mice at 24 h after LPS (20 mg/kg) and HVEM‐Fc or LTβR‐Fc injection

As a membrane‐anchored receptor for LIGHT, pretreatment with the soluble fusion proteins of the membrane‐anchored receptors HVEM‐Fc or LTβR‐Fc to block LIGHT‐HVEM or LIGHT‐LTβR interaction in mice reduced the BUN and SCr levels in LPS‐induced SA‐AKI (Figure 7A,B). Furthermore, in accordance with renal function, the LPS + HVEM‐Fc group and LPS + LTβR‐Fc group mice showed a milder injury compared to the WT group (Figure 7C,D). However, there were no significant differences between the LPS + HVEM‐Fc group and the LPS + LTβR‐Fc group in terms of pathological changes. These results further confirm that LIGHT aggravates SA‐AKI.

## 4 DISCUSSION

As a bidirectional immunoregulatory molecule, LIGHT has been reported to be involved in the pathogenesis of a variety of inflammatory and autoimmune diseases.^14^, ^15^, ^16^ However, the effect and underlying mechanisms of LIGHT in the pathogenesis of SA‐AKI remain poorly understood. In this study, mice with an intraperitoneal injection of LPS and HK‐2 cell challenged with LPS were employed as a model of SA‐AKI in vivo and in vitro, respectively. As a result, LIGHT deficiency was found to attenuate LPS‐induced SA‐AKI, and the relieved pathological damage was accompanied by a down‐regulated production of inflammatory mediators and inflammatory cell infiltration in LIGHT KO mice. The in vitro data further demonstrated that rhLIGHT protein promoted LPS‐treated HK‐2 cell injury. Moreover, mechanistic studies revealed that the LIGHT pathway promoted SA‐AKI by up‐regulating TLR4‐MyD88‐NFκB expression. Additionally, blocking LIGHT with HVEM‐Fc and LTβR‐Fc, two membrane‐anchored receptors soluble fusion protein for LIGHT, remarkably mitigated LPS‐induced SA‐AKI in vivo. Collectively, our data suggest that LIGHT aggravates LPS‐induced SA‐AKI via the TLR4‐MyD88‐NFκB pathway.

As a membrane‐anchored receptor for LIGHT, previous studies have shown that HVEM is mainly expressed on T cells, DC, or NK, including tumour and normal B lymphocytes,^30^, ^31^ whereas LTβR is mainly expressed in a variety of parenchyma and epithelial cells.^32^ Additionally, LIGHT and its receptors HVEM and LTβR are constitutively expressed in kidney tissues.^24^ In line with the literature, we found that LIGHT, HVEM and LTβR were expressed in kidney tissues. Intriguingly, in this study, we found for the first time that LIGHT was highly co‐localized with CK‐18, a marker of renal tubular epithelial cells. Given that renal tubular epithelial cells are the primary target of AKI,^23^ we speculated that the LIGHT pathway was closely associated with the pathogenesis of SA‐AKI. Previous research from our group has confirmed that LIGHT‐HVEM/LTβR is involved in IFNγ‐mediated MIN6 cell injury.^26^ Consistent with these findings, we further found that LIGHT blocking with soluble receptor fusion proteins HVEM‐Fc or LTβR‐Fc attenuated renal dysfunction and pathological injury in SA‐AKI. To the best of our knowledge, this study is the first to demonstrate that both receptors play an instrumental role in kidney disease, whereas previous studies have focused on the effect of LTβR activation.^33^

In addition, these data raised the fundamental question: by what mechanism does LIGHT aggravate LPS‐induced SA‐AKI? We speculated that there were two possible mechanisms. Firstly, LIGHT might indirectly mediate renal damage to aggravate SA‐AKI by promoting inflammatory responses. As a co‐stimulatory molecule, a myriad of research has demonstrated that LIGHT aggravates inflammatory responses and inflammatory‐related diseases.^14^, ^15^, ^16^ In line with these data, our study demonstrated that LIGHT deficiency led to a remarkable decrease in inflammatory mediator production and inflammatory cell infiltration in mice with SA‐AKI. Secondly, LIGHT might directly mediate renal damage to aggravate SA‐AKI. Renal tubular epithelial cells are a primary target of kidney injury and the progression of kidney disease.^23^ In the present study, LIGHT was found to be highly co‐localized with CK‐18, a marker of renal tubular epithelial cells, and the ultrastructural observation revealed the significant autophagy of tubular epithelial cells (data not shown). Moreover, exogenous LIGHT promoted LPS‐treated HK‐2 cell injury. These findings suggest that these mechanisms may collectively promote the pathogenesis of SA‐AKI to some extent.

**Figure 8:**
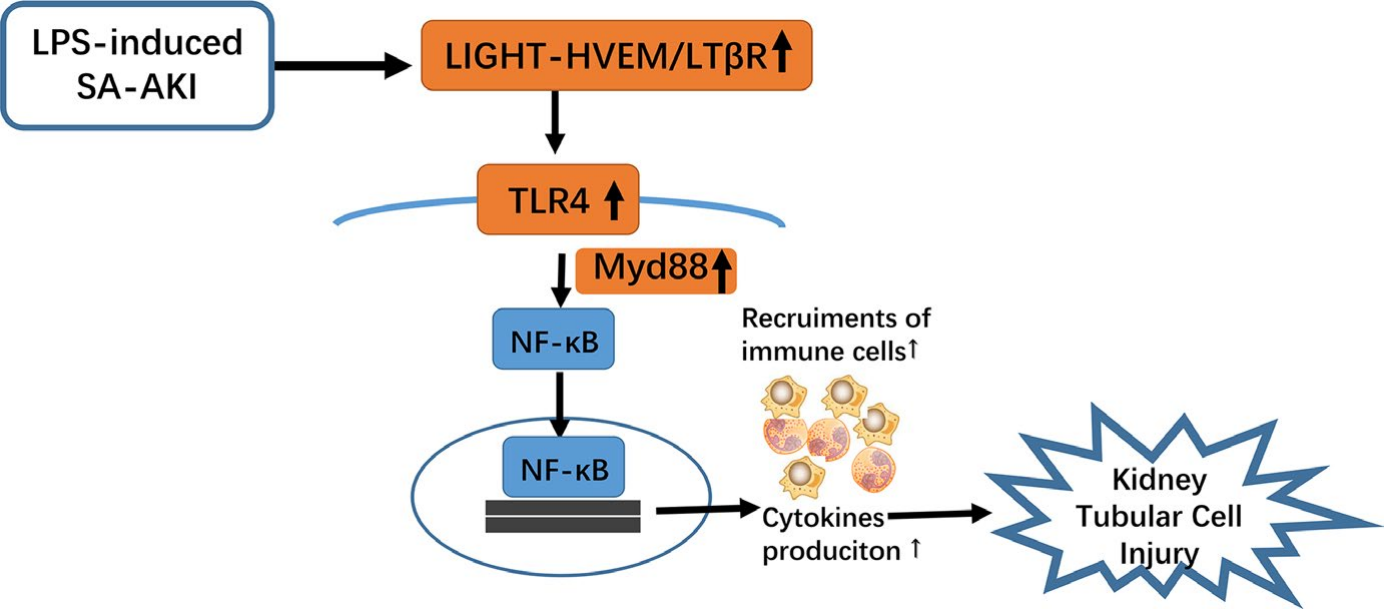
Schematic representation of the mechanism for LIGHT aggravating SA‐AKI

TLR4 plays a pivotal role in the pathogenesis of LPS‐induced SA‐AKI.^34^ Consistent with previous studies,^27^, ^28^, ^34^ we found that the expression of TLR4 and the downstream signalling molecules was significantly up‐regulated in the SA‐AKI model in vivo and in vitro. After the activation of TLRs, signals can be transmitted by two different signalling pathways: the MyD88‐dependent pathway and the TRIF‐dependent pathway.^29^ In addition, LPS induces the activation of the TLR4‐MyD88‐dependent signalling pathway, which contributes to the nuclear translocation and phosphorylation of NF‐κB.^35^ Our study showed that LIGHT deficiency significantly down‐regulated the levels of TLR4‐MyD88‐NF‐κB. TAK242, a selective TLR4 inhibitor, attenuated exogenous LIGHT‐induced HK‐2 cell injury and down‐regulated the expression of the TLR4‐MyD88‐NF‐κB pathway. These results suggest that the downstream signal of TLR4 may be activated through MyD88. In addition, we found that blocking LIGHT with an LTβR‐Fc or HVEM‐Fc fusion protein attenuated SA‐AKI. In contrast to our results, a previous study reported that LTβR activation induces TLR4 tolerance by combining with LTα1β2 ligand in vivo.^36^ Although seemingly contradictory, the findings of the previous study support our results: LPS increased LTβR activation, which can only bind with the LTα1β2 ligand if the LIGHT gene is knocked out. Therefore, LIGHT KO mice showed milder tissue injury and renal function and prolonged survival after LPS administration. Additionally, it has been previously demonstrated that LIGHT‐LTβR induced the activation of the NF‐κB pathway in a non‐canonical manner.^37^ Undoubtedly, LTβR costimulation synergistically improved the late NF‐κB reaction to TLR4 NF‐κB target gene‐expressions.^38^ However, recent studies have indicated that the classical NF‐κB activity has the ability to suppress non‐canonical NF‐κB signalling.^39^ Therefore, LIGHT‐induced TLR signalling was the main pathway to activate NF‐κB to mediate LPS‐induced SA‐AKI (Figure 8).

Taken together, our results demonstrate that LIGHT mediates SA‐AKI by promoting the TLR4‐MyD88‐NFκB signalling pathway. Our results provide the basis for a novel therapeutic strategy for the treatment of sepsis‐associated AKI in humans.

## CONFLICT OF INTEREST

All the authors declare no competing interests.

## AUTHOR CONTRIBUTION

**YU ZHONG:** Data curation (equal); Formal analysis (equal); Methodology (equal); Project administration (equal); Writing‐original draft (lead); Writing‐review & editing (equal). **Shun Wu:** Data curation (equal); Formal analysis (equal); Methodology (equal). **Yan Yang:** Data curation (equal). **Guiqing Li:** Data curation (equal). **Li Meng:** Data curation (equal). **Quan‐you Zheng:** Data curation (supporting); Funding acquisition (equal). **You Li:** Data curation (supporting). **Guilian Xu:** Resources (supporting); Writing‐review & editing (equal). **keqin zhang:** Conceptualization (equal); Funding acquisition (equal); Supervision (equal). **Kanfu Peng:** Conceptualization (equal); Funding acquisition (equal); Supervision (equal).

## Supporting information

Fig S1

## Data Availability

I confirm that my article contains a Data Availability Statement even if no data are available (list of sample statements) unless my article type does not require one (eg Editorials, Corrections, Book Reviews, etc).
