# Supplementary figures and images for: LIGHT aggravates sepsis‐associated acute kidney injury via TLR4‐MyD88‐NF‐κB pathway
Source: J Cell Mol Med. 2020 Sep 3;24(20):11936–48. doi: 10.1111/jcmm.15815 (PMC7579683; doi:10.1111/jcmm.15815)

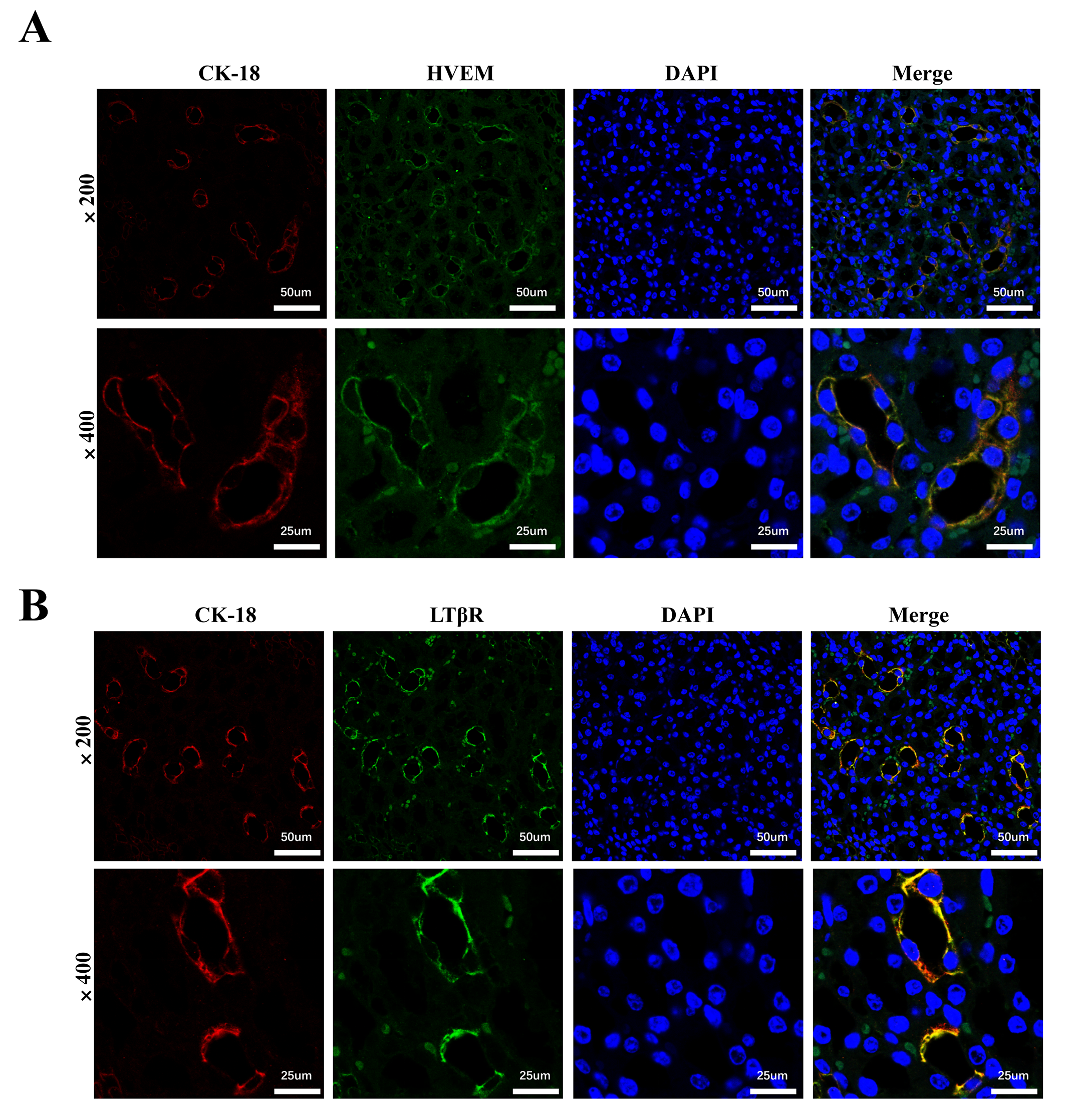

Supplement: Supplementary file 1 — Fig S1 [file JCMM-24-11936-s001.tif]
